# Supplementary material for: A Low-Cost, Thermostable, Cell-Free Protein Synthesis Platform for On-Demand Production of Conjugate Vaccines
Source: ACS Synth Biol. 2022 Dec 22;12(1):95–107. doi: 10.1021/acssynbio.2c00392 (PMC9872175; doi:10.1021/acssynbio.2c00392)
Supplement: Supplementary file 1 — sb2c00392_si_001.pdf [file sb2c00392_si_001.pdf]

A Low-Cost, Thermostable, Cell-free Protein Synthesis Platform for On-demand Production of Conjugate Vaccines

Katherine F. Warfel[a,b,c], Asher Williams[d], Derek A. Wong[a,b,c], Sarah E. Sobol[a,b,c], Primit Desai[e], Jie Li[f], Yung-Fu Chang[f], Matthew P. DeLisa[d,e,g], Ashty S. Karim[a,b,c], Michael C. Jewett[a,b,c,h,i,*]

Affiliations

[a]Department of Chemical and Biological Engineering, Northwestern University, 2145 Sheridan Road, Technological Institute E136, Evanston, IL 60208, USA
[b]Chemistry of Life Processes Institute, Northwestern University, 2170 Campus Drive, Evanston, IL 60208, USA
[c]Center for Synthetic Biology, Northwestern University, 2145 Sheridan Road, Technological Institute E136, Evanston, IL 60208, USA
[d]Robert Frederick Smith School of Chemical and Biomolecular Engineering, Cornell University, Ithaca, NY 14853 USA
[e]Biochemistry, Molecular & Cell Biology, Cornell University, Ithaca, NY 14853 USA
[f]Department of Population Medicine and Diagnostic Sciences, College of Veterinary Medicine, Cornell University, Ithaca, NY, 14853, USA
[g]Cornell Institute of Biotechnology, Cornell University, Ithaca, NY 14853 USA
[h]Robert H. Lurie Comprehensive Cancer Center, Northwestern University, 676 North Saint Clair Street, Suite 1200, Chicago, IL 60611, USA
[i]Simpson Querrey Institute, Northwestern University, 303 East Superior Street, Suite 11-131, Chicago, IL 60611, USA

[*]To whom correspondence should be addressed
Michael C. Jewett
Phone: 1-847-497-5007
Email: m-jewett@northwestern.edu

Supporting Information

Supporting Figures 1-11
Supporting Tables 1-5
Supporting References

Supplementary Figures

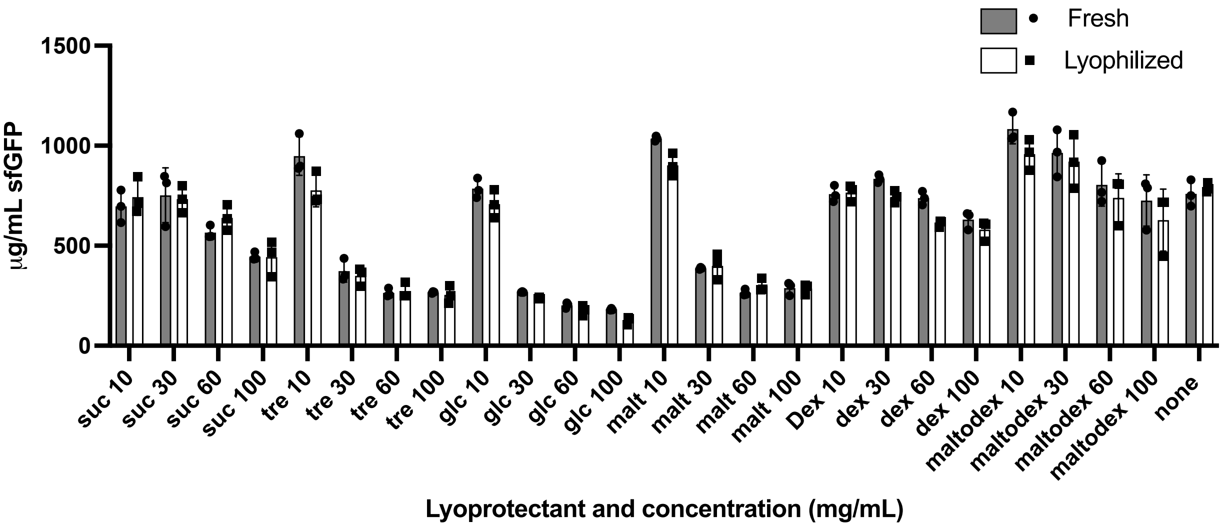

Figure S1. Impact of lyoprotectant additives on fresh (un-lyophilized) and lyophilized (un-stored/zero-week timepoint) CFE PEP formulation reactions using BL21 Star (DE3) extract. The impact of sucrose (suc), trehalose (tre), glucose (glc), maltose (malt), dextran (dex), and maltodextrin (maltodex) at 0, 10, 30, 60, 100 mg/mL final concentration on fresh (grey bars) and lyophilized (white bars) CFE reaction productivity after 20 hours. Control with no lyoprotectant (none) is shown on the far right. Lyophilized reactions were rehydrated immediately after lyophilization as an "un-stored" control. Error bars represent standard deviation of three CFE reaction replicates (n=3).

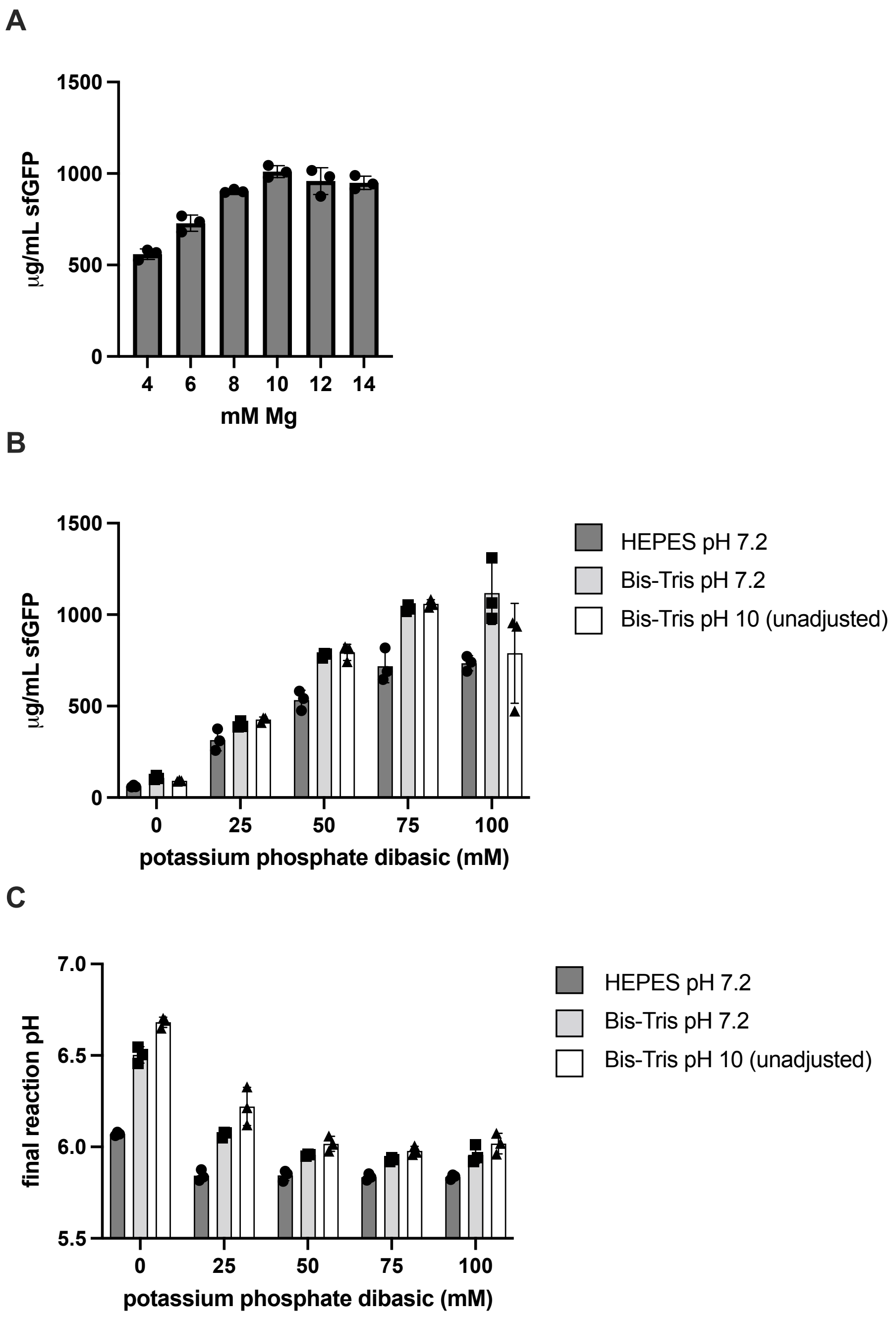

Figure S2. Optimization of CFE reagents in fresh (un-lyophilized) reactions for MD formulation in BL21 Star (DE3) extract. (A) Magnesium optimization of lysate using PEP as an energy source with sfGFP synthesis as a reporter. (B) Using 10 mM Mg^{2+} in the MD reaction formulation, sfGFP production was used to determine the optimal concentration of potassium phosphate dibasic (pH 7.2) in the CFE reaction to activate maltodextrin metabolism. Impact of additional buffer to stabilize pH in the MD formulation was tested using 57 mM of either HEPES with pH adjusted to 7.2 (dark grey), Bis-Tris with pH adjusted to 7.2 (light grey), or Bis-Tris with unadjusted pH (pH 10) (white). (C) Impact of conditions in B on final pH of the cell-free protein expression reaction, measured after 20 hours of sfGFP synthesis at 30 °C. Reactions were not lyophilized. Error bars represent standard deviation of three CFE reaction replicates (n=3).

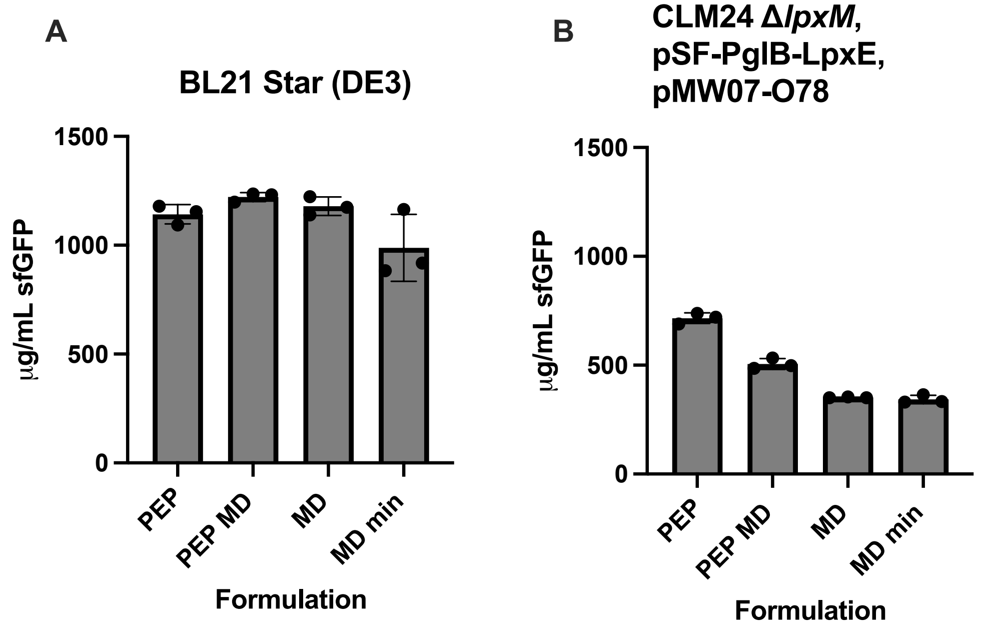

Figure S3. sfGFP yields of four formulations in fresh (un-lyophilized) CFE reactions using different extracts. (A) sfGFP synthesis in CFE reactions using BL21 Star (DE3) extract for each formulation. (B) sfGFP synthesis in CFE reactions using the iVAX strain (CLM24 Δ*lpxM* with overexpression of glycosylation machinery from pSF-PglB-LpxE and pMW07-O78 plasmids) extract for each formulation. Reactions were not lyophilized. Error bars represent standard deviation of three CFE reaction replicates (n=3).

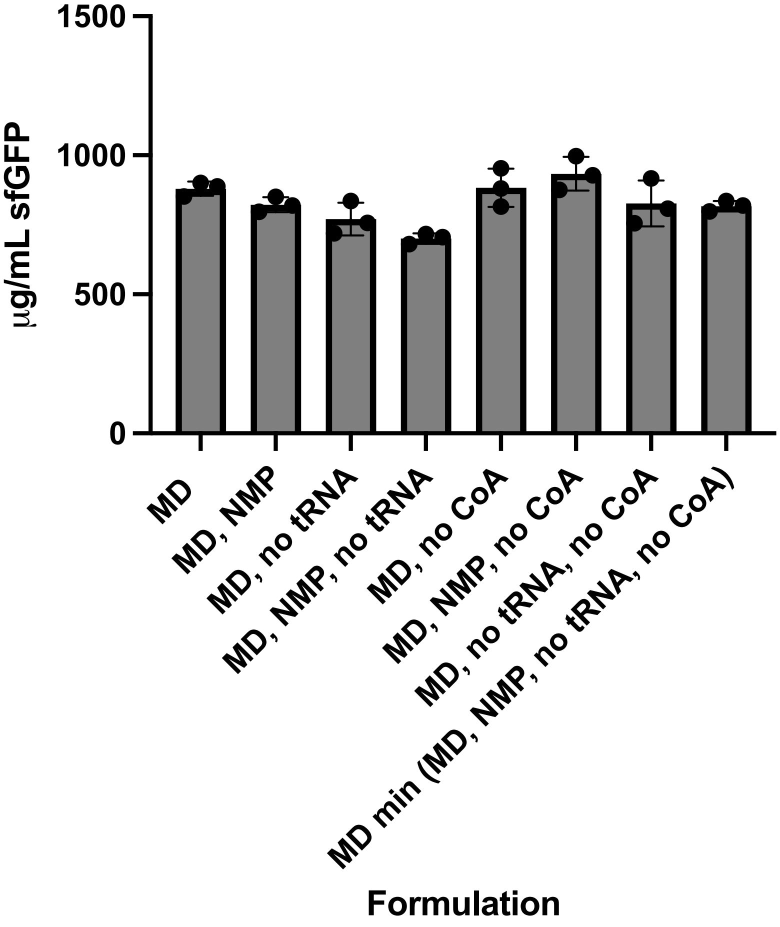

Figure S4. sfGFP yields in fresh (un-lyophilized) CFE reactions using BL21 Star (DE3) extract showing the impact of the formulation changes made to the MD formulation to arrive at the MD min formulation. sfGFP yields in a BL21 Star (DE3) extract for the MD formulation (far left) and the impact of changing NTPs to NMPs, removing tRNA, and removing CoA, independently and in combination. sfGFP yields in the MD min formulation (all changes in the same reaction) are displayed on the far right. Reactions were not lyophilized. Error bars represent standard deviation of three CFE reaction replicates (n=3).

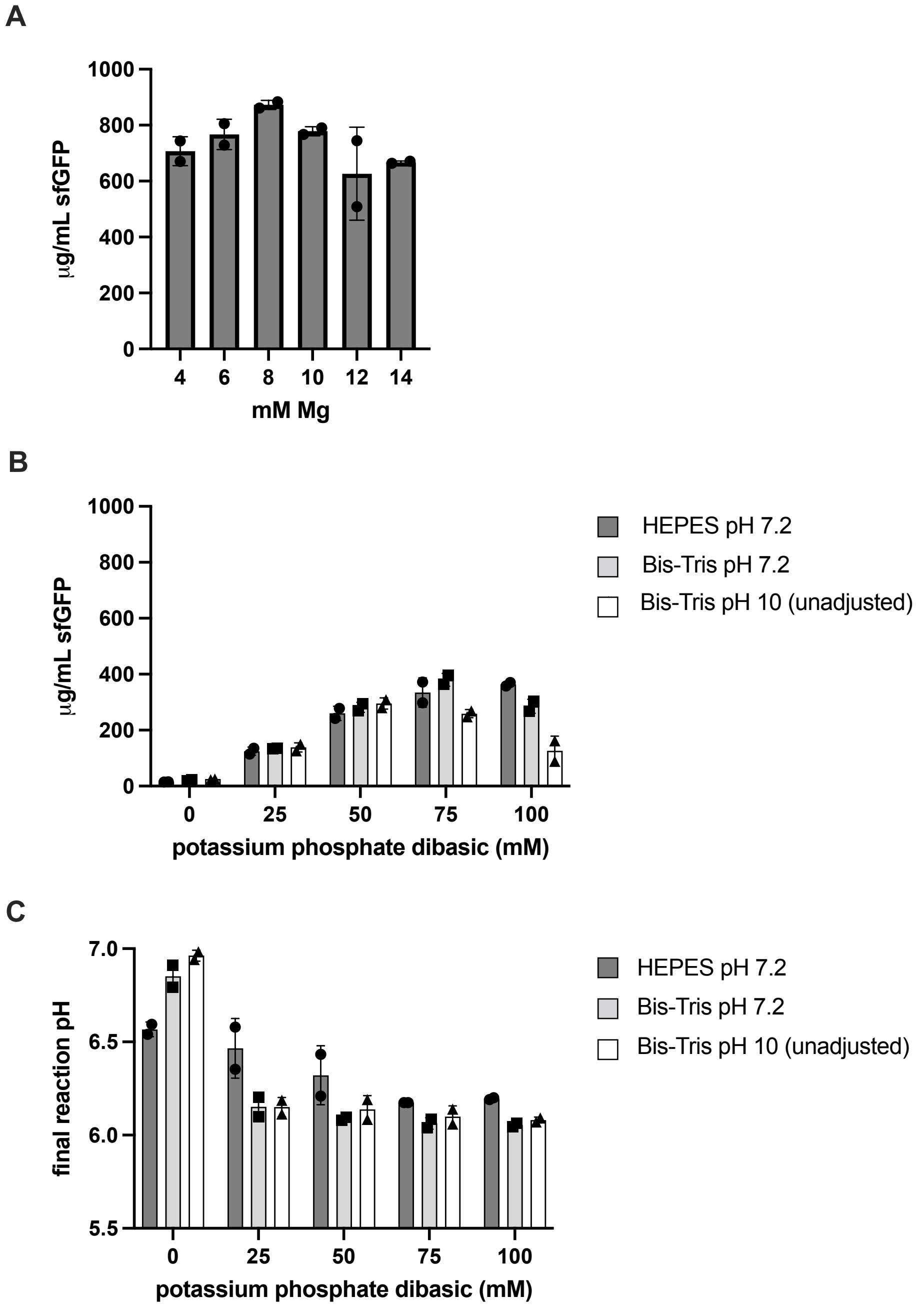

Figure S5. Optimization of CFE reagents for fresh (un-lyophilized) CFE reactions with the MD formulation in iVAX extract (CLM24 Δ*lpxM* with overexpression of glycosylation machinery from pSF-PglB-LpxE and pMW07-O78 plasmids). (A) Magnesium optimization of lysate using PEP as an energy source and sfGFP synthesis as a readout. (B) Using 8 mM Mg^{2+} in the MD reaction formulation, sfGFP production was used to determine the optimal concentration of potassium phosphate dibasic (pH 7.2) in the CFE reaction to activate maltodextrin metabolism. Impact of additional buffer to stabilize pH in the MD formulation was tested using 57 mM of either HEPES with pH adjusted to 7.2 (dark grey), Bis-Tris with pH adjusted to 7.2 (light grey), or Bis-Tris with unadjusted pH (pH 10) (white). (C) Impact of conditions in B on final pH of the cell-free reaction, measured after 20 hours of sfGFP synthesis at 30 °C. Reactions were not lyophilized. Error bars represent average error of two CFE reaction replicates (n=2).

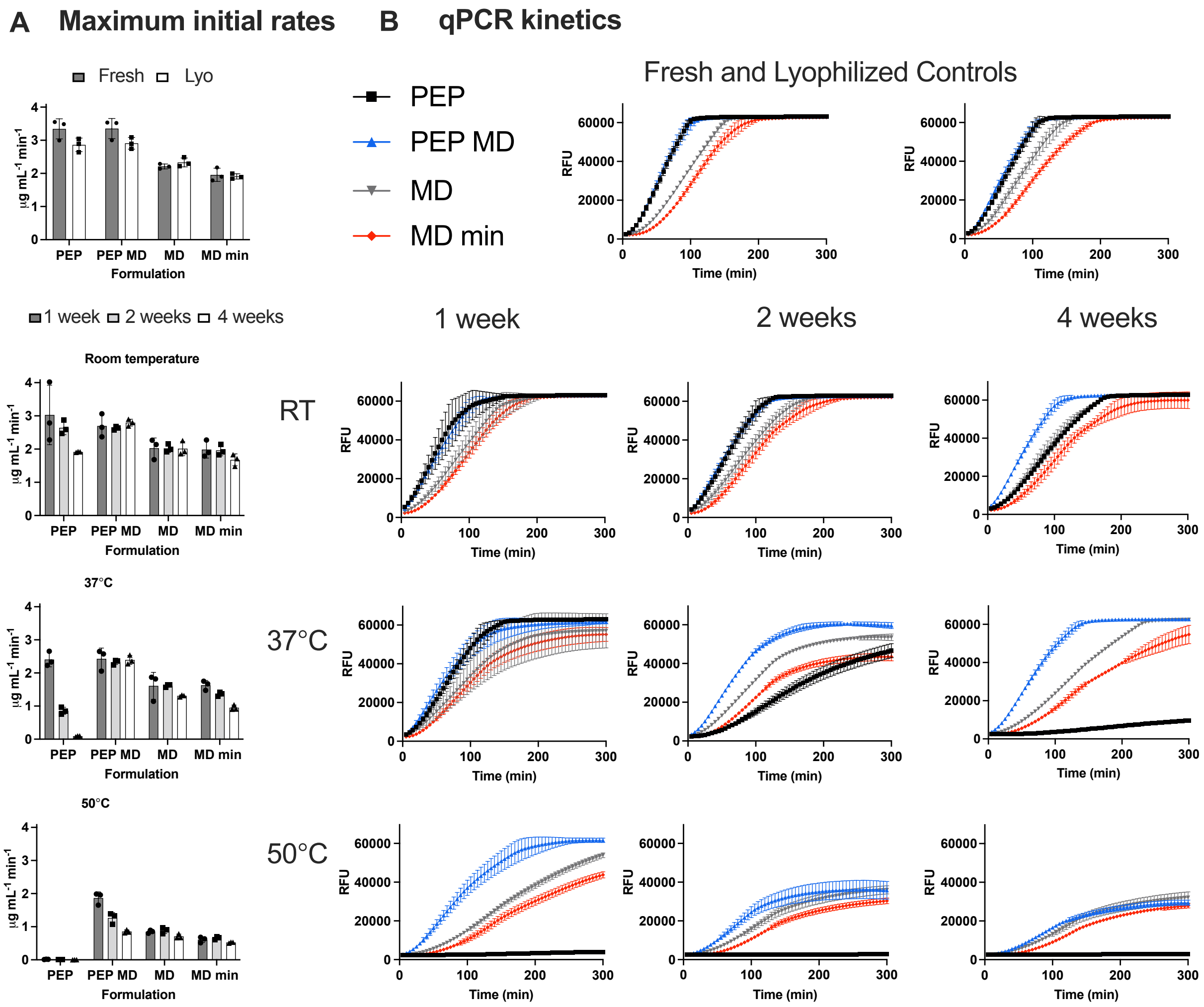

Figure S6. Rates of sfGFP synthesis in all formulations using the iVAX extract. (A) Maximum initial rates of reactions were calculated over the first 90 minutes of protein synthesis using qPCR measurement of fluorescence every 5 minutes. Fluorescence was converted from RFU to µg/mL sfGFP using standard curves with ^{14}C-labeled sfGFP. (B) qPCR traces recording fluorescence every 5 minutes for all reaction conditions measured in RFUs over the initial 300 minutes of the reaction. While reactions were incubated in the instrument at 30 °C for 20 hours before endpoint sfGFP values were read, the instrument reached the limit of detection at ~60,000 RFU, thus only initial kinetic data is shown. Maximum reached at ~60,0000 RFU is due to limit of detection of the instrument and is not representative of protein synthesis levels at that timepoint. Error bars represent standard deviation of three CFE reaction replicates (n=3). Fresh (un-lyophilized) reactions, lyophilized (un-stored reactions), and reactions stored for 1, 2, and 4 weeks at either room temperature, 37 °C, or 50 °C were analyzed.

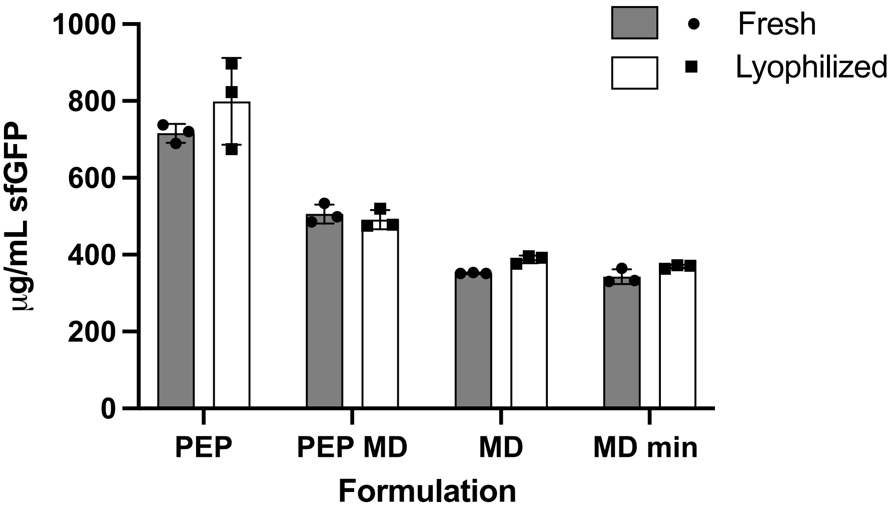

Figure S7. sfGFP yields of fresh (un-lyophilized) and lyophilized (un-stored/zero-week timepoint) controls of all CFE reaction formulations with the iVAX extract. sfGFP yields of 5 μL fresh reactions that were not lyophilized (grey) and lyophilized reactions that were un-stored (white) after 20 hours of incubation at 30 °C using the iVAX extract. Error bars represent standard deviation of three CFE reaction replicates (n=3).

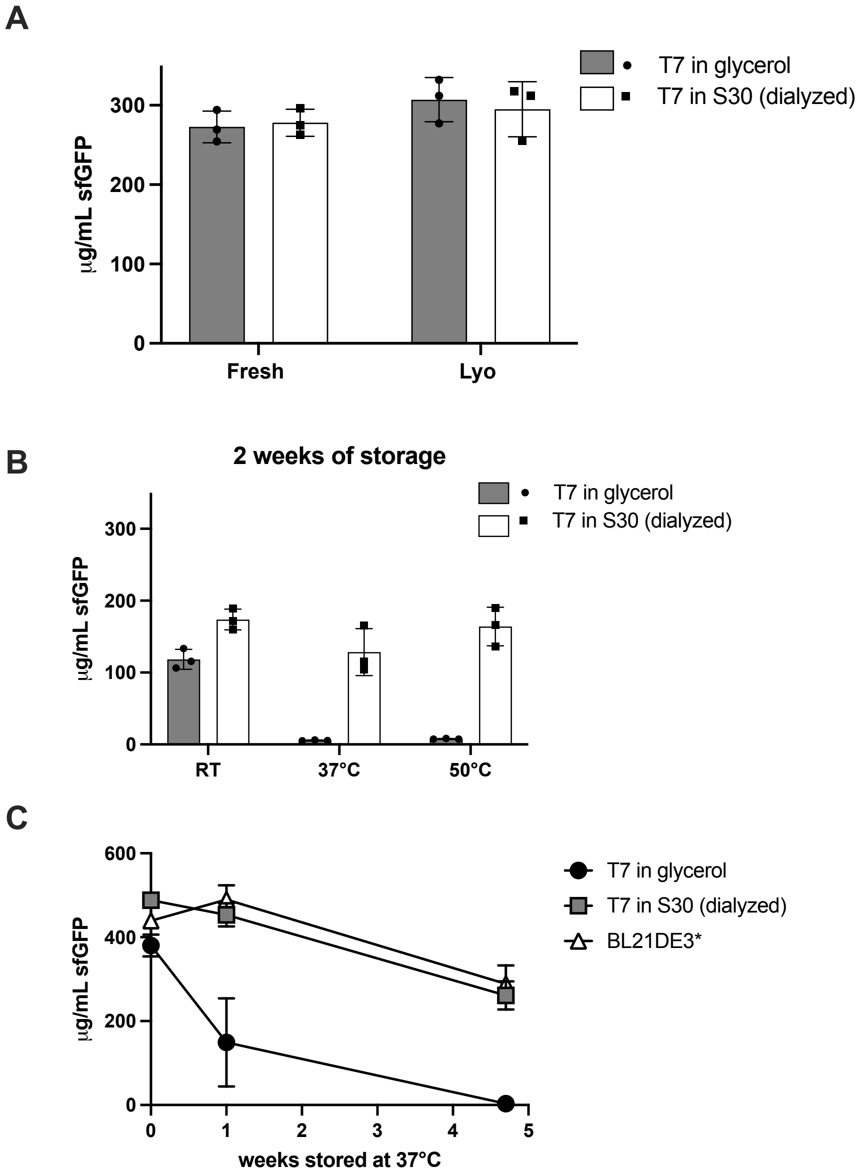

Figure S8. Impact of glycerol (contained in purified T7) on MD formulation. (A) sfGFP yields of 5 µL fresh (un-lyophilized) reactions and lyophilized (un-stored/zero-week timepoint) reactions after 20 hours of incubation at 30 °C using the MD formulation in the iVAX extract. Grey bars contain T7 source in 50% glycerol and white bars have the same original T7 source dialyzed into S30 buffer. (B) Reactions from the same experiment are shown in A after storage at either room temperature (RT), 37 °C, or 50 °C for 2 weeks. Reactions containing glycerol from the T7 stock are shown in grey while reactions with no glycerol using the dialyzed T7 stock are shown in white. (C) Endpoint sfGFP synthesis timecourse from MD formulation CFE reactions with T7 source as either T7 in glycerol (black circle), T7 in S30 buffer, dialyzed from glycerol (grey squares), or supplementing the reaction with a final concentration of 3.3% v/v BL21 Star (DE3) extract that had T7 overexpressed in the strain before lysis (white triangles). Lysate for this experiment was derived from the parental CLM24 strain not modified for glycosylation or remodeled endotoxin. Reaction conditions optimized for this strain were 8 mM Mg^{2+}, 50 mM phosphate, and Bis-Tris (pH 10). Error bars represent standard deviation of three CFE reaction replicates for A, B, and C (n=3).

A

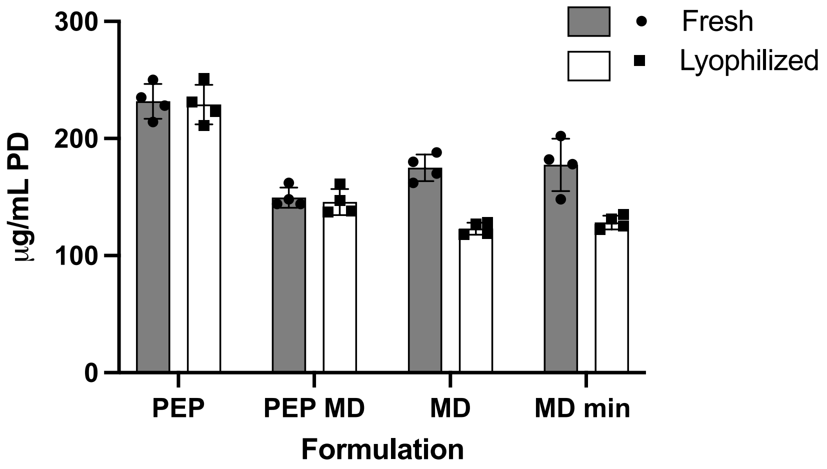

B

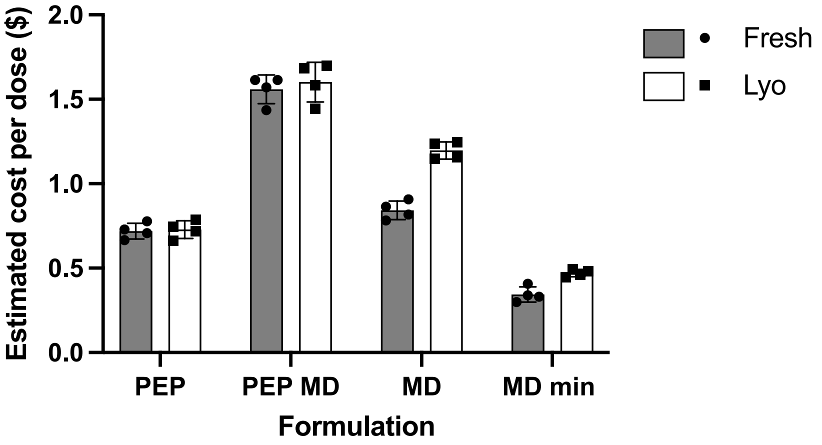

Figure S9. PD yields and estimated cost per conjugate vaccine dose of fresh (un-lyophilized) and lyophilized (un-stored) controls of all CFE reaction formulations with the iVAX extract. (A) PD yields of 15 µL fresh (un-lyophilized) reactions (grey) and lyophilized (un-stored/ zero-week timepoint) reactions (white) after 20 hours of incubation at 30 °C using the iVAX extract. Yields were measured using ^{14}C-leucine incorporation. (B) Estimated cost per dose of conjugate vaccine obtained from fresh (un-lyophilized) (grey) and lyophilized (un-stored) (white) iVAX reactions. Calculations consider estimated % glycosylation as measured by densitometry in **Figure S10D** and assume a 24 µg dose. Error bars represent standard deviation of four CFE reaction replicates (n=4).

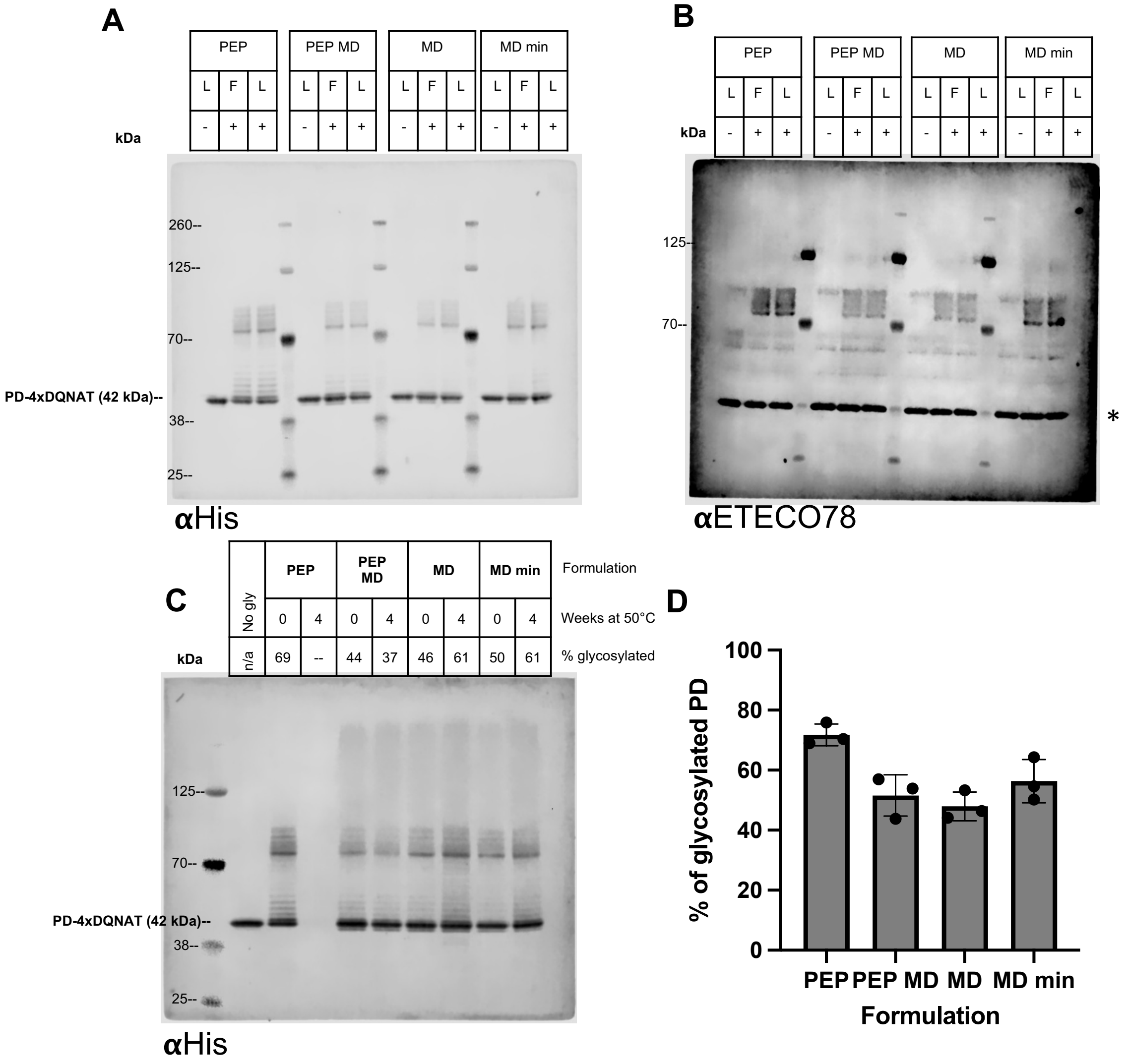

Figure S10. Glycosylation of PD with ETEC-O78 O-antigen in iVAX reactions. (A) Anti-His Western blot against His-tagged carrier protein (PD) demonstrating glycosylation with the ETEC O78 O-antigen in fresh (un-lyophilized) and lyophilized (un-stored/zero-week timepoint) reactions. From left to right, three control reactions are shown for each formulation (PEP, PEP MD, MD, and MD min). First a negative lyophilized (un-stored) control (aglycosylated PD) using an iVAX lysate with no ETEC-O78 expression is denoted as (L, -). Then, a fresh (un-lyophilized) control in the iVAX lysate (glycosylated PD) is denoted as (F, +). Finally, a lyophilized (un-stored) control in the iVAX lysate (glycosylated PD) is shown and denoted as (L, +). An equal concentration of PD as determined by ^{14}C-leucine incorporation was loaded in each well. Each formulation is separated by a Chameleon 800 ladder annotated on the left-hand side of the blot. (B) Anti-ETEC-O78 glycan western blot demonstrating glycosylation of PD with ETEC-O78 O-antigen in fresh and lyophilized controls. Sample orientation is the same as in A. O-antigen banding is visible between the 70-kDa and 125-kDa MW markers. The band annotated with an asterisk on the left of the blot is a contaminating band found in both glycosylated and unglycosylated samples also observed in our previous work[1]. An equal concentration of PD as determined by ^{14}C-leucine incorporation was loaded in each well. Each formulation is separated by a Chameleon 800 ladder annotated on the

left-hand side of the blot. (C) Uncropped Anti-His western blot shown in **Figure 4E** demonstrating glycosylation of PD with ETEC-O78 O-antigen in samples stored for 0 weeks (lyophilized, un-stored) and samples stored for 4 weeks at 50 °C. The first lane has a lyophilized (un-stored) negative glycosylation control reaction for the PEP formulation using an iVAX lysate with no ETEC-O78 expression. Then from left to right for each formulation PEP, PEP MD, MD, and MD min, there is a lyophilized sample stored for 0 weeks (lyophilized, un-stored) and then a sample that was stored for 4 weeks at 50 °C. An equal concentration of PD as determined by ^{14}C-leucine incorporation was loaded in each well. For the PEP formulation after 4 weeks of storage at 50 °C, no protein synthesis was detected, so the volume equivalent to that used for the least concentrated sample was run on the gel as a verification. Percent glycosylation as estimated by densitometry with Image Studio Lite (Licor) software for each lane is reported above the blot and used for glycoprotein calculation in main **Figure 4D**. (D) Percent of glycosylated PD (glycosylated PD/total PD) was estimated for each formulation using densitometry and Image Studio Lite (Licor) software. Error bars represent standard deviation of three CFE reactions (n=3). Triplicate values include % glycosylation from fresh and lyophilized samples from A and lyophilized samples from C for each formulation. Gels are representative of three independent experiments. Values were used for cost calculation in main Figure 4F.

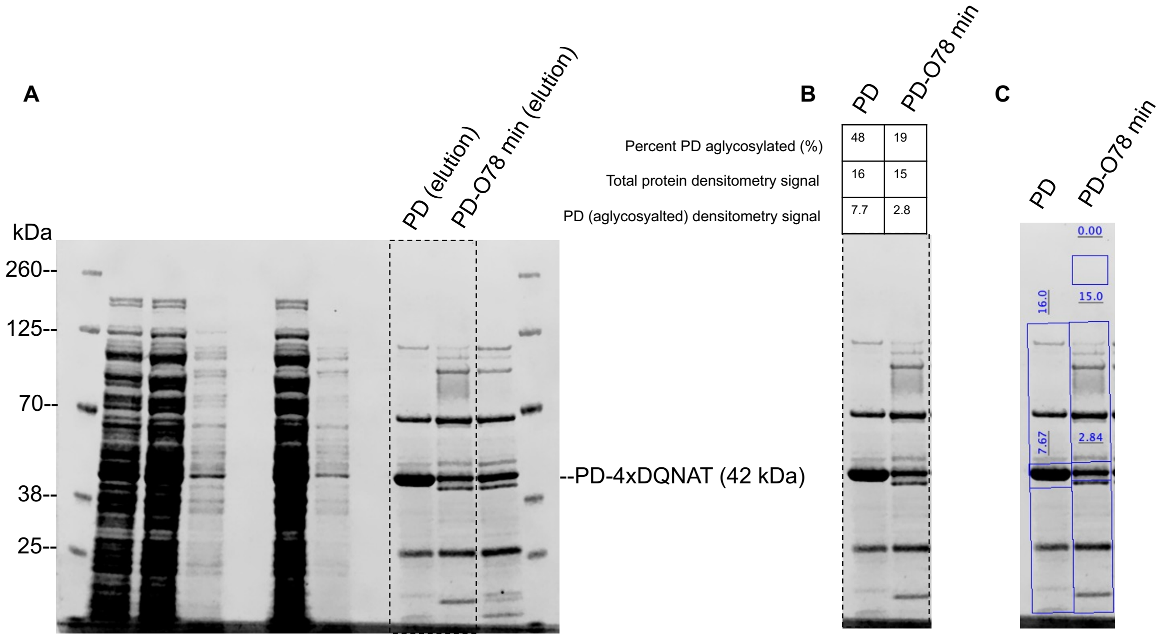

Figure S11. Analysis of purified cell-free derived protein for mouse study. (A) Uncropped purification analysis including elution fractions of PD (negative control) and PD-O78 in the MD min formulation used for determination of conjugate concentration. The two lanes containing the purified fractions are boxed. (B) Cropped lanes containing elution fractions of PD (negative control) and PD-O78 from A. Table above the gel displays the total protein densitometry signal and PD (aglycosylated) densitometry signal as determined by Licor ImageStudio. Table also includes approximate percent purity of the aglycosylated PD in the sample as determined by (PD (aglycosylated) densitometry signal/ total protein densitometry signal). (C) Cropped lanes containing elution fractions of PD (negative control) and PD-O78 from A and B with the overlayed boxes that were used for densitometry determination of purity. The small box in the top right-hand corner represents the area used for background subtraction.

Supplementary Tables

Table S1. Cost breakdown of the CFE reaction formulations used in this work. Cost per liter of CFE reaction for all reagents present in the PEP, PEP MD, MD, and MD min formulations. This table is based on BL21 Star (DE3) extract as is presented in **Figure 2** of the main text.

Component	PEP ($/L)	PEP MD ($/L)	MD ($/L)	MD min ($/L)
magnesium glutamate	0.94	0.94	0.94	0.94
ammonium glutamate	5.97	5.97	5.97	5.97
potassium glutamate	8.48	8.48	8.48	8.48
ATP	15.16	15.16	15.16	
CTP	241.05	241.05	241.05	
UTP	277.74	277.74	277.74	
GTP	320.63	320.63	320.63	
AMP				6.91
CMP				8.86
UMP				5.85
GMP				3.70
folinic acid	22.62	22.62	22.62	22.62
tRNA	255.00	255.00	255.00	
amino acids	100.54	100.54	100.54	100.54
PEP	2065.42	2065.42		
maltodextrin		19.80	19.80	19.80
NAD	110.39	110.39	110.39	110.39
CoA	435.19	435.19	435.19	
oxalic acid	0.19	0.19	0.19	0.19
putrescine	0.74	0.74	0.74	0.74
spermidine	5.35	5.35	5.35	5.35
HEPES	6.06	6.06		
Bis-Tris			9.77	9.77
potassium phosphate dibasic			3.18	3.18
plasmid DNA	333.25	333.25	333.25	333.25
extract	728.25	728.25	728.25	728.25
**Total $/L CFE reaction**	**4932.97**	**4952.77**	**2894.24**	**1374.79**
**Total $/mL CFE reaction**	**4.93**	**4.95**	**2.89**	**1.37**

Table S2. Cost breakdown of cell extract. Note that the base extract cost is used in all calculations in this work to make claims more generalizable, as variable component costs are approximately the same for both strains used in this study and are dependent on strain and plasmid used to make extract. Assumptions used in calculations are listed below.

Assumptions:

1. 4 mL of extract are produced per liter cell culture.
2. 30% v/v extract is used in CFE reactions.
3. Only raw materials added to cell culture are considered for a base case of extract without variable components such as inducers or antibiotics.
4. Labor costs associated with extract production are not considered.
5. Equipment costs are not considered.

**Compound Name**	**Vendor**	**Catalog Number**	**$/g**	**$/L culture**
**Constant components**				
tryptone	Sigma	T7293-1kg	0.272	4.352
yeast extract	Sigma	Y1625-1kg	0.246	2.46
sodium chloride	Sigma	S3014-5kg	0.0438	0.219
potassium phosphate, monobasic	Sigma	P9791-1kg	0.168	0.504
potassium phosphate, dibasic	Sigma	60353-1kg	0.312	2.184
**Variable components**				
glucose	Sigma	G8270-5kg	0.024	0.432
IPTG (0.5 mM)	Sigma	I6758-10g	49.2	5.86218
arabinose (0.02 wt% in media)	Sigma	A3256-1kg	1.77	0.354
carbenicillin disodium salt	Sigma	C1389-10g	70.7	7.07
chloramphenicol	Sigma	C0378-100g	1.66	0.05644

**Extract**	**$/L cells**	**$/mL extract**	**$/mL CFE reaction**	**$/L CFE reaction**
**Base**	9.71	2.4275	0.72825	728.25
**BL21 Star (DE3)**	16	4	1.2	1200
**iVAX**	17.19	4.2975	1.28925	1289.25

Table S3. Information on all reagents added to the CFE reaction. Costs are recorded as of January 2022 at lab scale from vendors used in this work**.**

Compound Name	Vendor	Catalog Number	$/g	$/L reaction
**For PEP formulation**				
magnesium glutamate (10 mM)	Sigma	49605-250g	0.242	0.94
ammonium glutamate	Biosynth	FG28929-.1kg	3.6383	5.97
potassium glutamate	Sigma	G1501-1kg	0.321	8.48
ATP	Sigma	A2383-25G	22.92	15.16
CTP	Sigma	C1506-1g	538	241.05
UTP	Sigma	U6625-1g	594	277.74
GTP	Sigma	G8877-1g	721	320.63
folinic acid	Sigma	47612-1g	754	22.62
tRNA	Sigma	10109550001-.5g	1500	255.00
amino acids (cost for 1 g of each)	Sigma	LAA21-1kt	457	100.54
PEP	Sigma	10108294001-1g	334	2065.42
NAD	Sigma	N8535-15VL	416	110.39
CoA	Sigma	C3144-1g	2100	435.19
oxalic acid	Sigma	P0963-500g	0.26	0.19
putrescine	Sigma	P5780-25g	4.6	0.74
spermidine	Sigma	S2626-25g	24.56	5.35
HEPES	Sigma	H3375-5kg	0.446	6.06
Plasmid DNA	Zymo	D4201-50 preps	25000	333.25
**For modified formulations**				
AMP	Sigma	01930-25g	14.72	6.91
CMP	Sigma	C1006-5g	28.4	8.86
UMP	Sigma	U6375-10g	18.7	5.85
GMP	Sigma	G8377-100g	10.7	3.70
maltodextrin	Sigma	419672-500g	0.33	19.80
Bis-Tris	Sigma	B9754-1kg	0.819	9.77
potassium phosphate, dibasic	Sigma	60353-1kg	0.312	3.18
**For iVAX reactions**				
DDM	Anatrace	D310S-25g	39.56	39.56
$MnCl_2$	Sigma	221279-500G	0.272	1.35

Table S4. Components of the CFE reaction formulations used in this work. Final concentration of each reagent used in the CFE reaction for all formulations is provided in mM unless otherwise noted in the table. Cells filled in grey indicate that a component is not present in the reaction formulation described by that column. *Choice of buffer is extract source strain dependent for the MD and MD min formulations, but both buffers were used at the same final concentration.

Component	PEP	PEP MD	MD	MD min
magnesium glutamate	10	10	10	10
ammonium glutamate	10	10	10	10
potassium glutamate	130	130	130	130
ATP	1.2	1.2	1.2	
CTP	0.85	0.85	0.85	
UTP	0.85	0.85	0.85	
GTP	0.85	0.85	0.85	
AMP				1.2
CMP				0.85
UMP				0.85
GMP				0.85
folinic acid	0.03 mg/mL	0.03 mg/mL	0.03 mg/mL	
tRNA	0.17 mg/mL	0.17 mg/mL	0.17 mg/mL	
amino acids	2	2	2	2
PEP	30	30		
maltodextrin		60 mg/mL	60 mg/mL	60 mg/mL
NAD	0.4	0.4	0.4	0.4
CoA	0.27	0.27	0.27	
oxalic acid	4	4	4	4
putrescine	1	1	1	1
spermidine	1.5	1.5	1.5	1.5
HEPES/Bis-Tris*	57	57	57	57
potassium phosphate dibasic			75	75
plasmid DNA	13.33 ng/μL	13.33 ng/μL	13.33 ng/μL	13.33 ng/μL
extract	30 % v/v	30 % v/v	30 % v/v	30 % v/v

Table S5. Strains and plasmids used in this study.

Strain or Plasmid	Description
BL21 Star (DE3)	*E. coli* B strain for expression.
CLM24 Δ*lpxM*[1] (Addgene 132780)	*E. coli* K-12 strain CLM24 with a knockout of the aceyltransferase *LpxM* to alter endotoxin structure.
pJL1-sfGFP[2] (Addgene 102634)	sfGFP variant with a C-terminal strep tag in the pJL1 expression vector.
pJL1-PD-4x DQNAT[1] (Addgene 128391)	*H. influenzae* protein D modified with a C-terminal 4x DQNAT glycosylation sequon and a 6x His tag, recognized by *C. jejuni* PglB in the pJL1 expression vector.
pMW07-O78[1,3,4]	*E. coli* O78 O-antigen gene cluster in the pMW07 expression vector.
pSF-PglB-LpxE[1] (Addgene 128389)	*C. jejuni* PglB with a C terminal LpxE phosphatase from *F. tularensis* and a 1x-FLAG tag in the pSF expression vector.

Supporting References

(1) Stark, J. C.; Jaroentomeechai, T.; Moeller, T. D.; Hershewe, J. M.; Warfel, K. F.; Moricz, B. S.; Martini, A. M.; Dubner, R. S.; Hsu, K. J.; Stevenson, T. C.; Jones, B. D.; DeLisa, M. P.; Jewett, M. C. On-Demand Biomanufacturing of Protective Conjugate Vaccines. *Sci Adv* **2021**, *7* (6). https://doi.org/10.1126/sciadv.abe9444.

(2) Stark, J. C.; Huang, A.; Nguyen, P. Q.; Dubner, R. S.; Hsu, K. J.; Ferrante, T. C.; Anderson, M.; Kanapskyte, A.; Mucha, Q.; Packett, J. S.; Patel, P.; Patel, R.; Qaq, D.; Zondor, T.; Burke, J.; Martinez, T.; Miller-Berry, A.; Puppala, A.; Reichert, K.; Schmid, M.; Brand, L.; Hill, L. R.; Chellaswamy, J. F.; Faheem, N.; Fetherling, S.; Gong, E.; Gonzalzles, E. M.; Granito, T.; Koritsaris, J.; Nguyen, B.; Ottman, S.; Palffy, C.; Patel, A.; Skweres, S.; Slaton, A.; Woods, T.; Donghia, N.; Pardee, K.; Collins, J. J.; Jewett, M. C. BioBits^{TM} Bright: A Fluorescent Synthetic Biology Education Kit. *Sci Adv* **2018**, *4* (8), eaat5107. https://doi.org/10.1126/sciadv.aat5107.

(3) Çelik, E.; Ollis, A. A.; Lasanajak, Y.; Fisher, A. C.; Gür, G.; Smith, D. F.; DeLisa, M. P. Glycoarrays with Engineered Phages Displaying Structurally Diverse Oligosaccharides Enable High-Throughput Detection of Glycan-Protein Interactions. *Biotechnol. J.* **2015**, *10* (1), 199–209. https://doi.org/10.1002/biot.201400354.

(4) Chen, L.; Valentine, J. L.; Huang, C.-J.; Endicott, C. E.; Moeller, T. D.; Rasmussen, J. A.; Fletcher, J. R.; Boll, J. M.; Rosenthal, J. A.; Dobruchowska, J.; Wang, Z.; Heiss, C.; Azadi, P.; Putnam, D.; Trent, M. S.; Jones, B. D.; DeLisa, M. P. Outer Membrane Vesicles Displaying Engineered Glycotopes Elicit Protective Antibodies. *Proc. Natl. Acad. Sci. U. S. A.* **2016**, *113* (26), E3609-18. https://doi.org/10.1073/pnas.1518311113.
